# Feasibility of a brief intervention delivered via mobile phone to reduce harmful drinking and injury among trauma patients in New Zealand

**DOI:** 10.1186/1940-0640-7-S1-A92

**Published:** 2012-10-09

**Authors:** Shanthi Ameratunga, Emily Smith, Bridget Kool, Kimiora Raerino

**Affiliations:** 1 Section of Epidemiology and Biostatistics, School of Population Health, University of Auckland, New Zealand; 2 School of Population Health, University of Auckland, Auckland, New Zealand

## 

Building on the success of the Stop Smoking Over Mobile Phone (STOMP) trial and the Social Cognitive Theory-Based Intervention (STUB-IT) trial for smoking cessation, this pilot study examined the feasibility of brief intervention (BI) delivered by mobile-health technology to reduce problem drinking and injury among trauma patients presenting to the hospital. We interviewed 30 Maori, Pacific Island, and Pakeha (European/white) patients to explore their perceptions of the proposed mobile-phone–delivered intervention and the barriers and facilitators to participation and compliance. Participants were highly supportive of the intervention concept, noting aspects of message content and delivery that would appeal to the major ethnic communities as well as potential barriers that required attention. Text messages (informed by cognitive-behavioral and social-learning theory) that were adapted for the cultural context had particular appeal, as was the proposed delivery of one or two motivational messages on Fridays/weekends over a period of up to four weeks following hospital discharge. Collecting injury-outcome data through record linkage to national databases of hospital discharges and claims to New Zealand’s universal fully-funded accident insurance scheme was both feasible and effective. The proposed trial of 6000 participants appears feasible and acceptable to patients from communities of interest, with economies of scale in both the implementation of the intervention and trial methodology. If demonstrated to be effective, this approach to BI has the potential to be cost-effective, highly scalable, and accessible to harder-to-reach communities in New Zealand and elsewhere. As shown in previous trials, this strategy could also reduce ethnic and socioeconomic inequalities in access to interventions.

